# Predicting diagnostic gene biomarkers in patients with diabetic kidney disease based on weighted gene co expression network analysis and machine learning algorithms

**DOI:** 10.1097/MD.0000000000035618

**Published:** 2023-10-27

**Authors:** Qian Gao, Huawei Jin, Wenfang Xu, Yanan Wang

**Affiliations:** a Affiliated Hospital of Shaoxing University of Edocrine and Metabolism Department, Zhejiang, China; b Affiliated Hospital of Shaoxing University of Clinical Laboratory, Zhejiang, China.

**Keywords:** diabetic kidney disease, machine-learning algorithms, weighted gene co-expression network analysis

## Abstract

The present study was designed to identify potential diagnostic markers for diabetic kidney disease (DKD). Two publicly available gene expression profiles (GSE142153 and GSE30528 datasets) from human DKD and control samples were downloaded from the GEO database. Differentially expressed genes (DEGs) were screened between 23 DKD and 10 control samples using the gene data from GSE142153. Weighted gene co expression network analysis was used to find the modules related to DKD. The overlapping genes of DEGs and Turquoise modules were narrowed down and using the least absolute shrinkage and selection operator regression model and support vector machine-recursive feature elimination analysis to identify candidate biomarkers. The area under the receiver operating characteristic curve value was obtained and used to evaluate discriminatory ability using the gene data from GSE30528. A total of 110 DEGs were obtained: 64 genes were significantly upregulated and 46 genes were significantly downregulated. Weighted gene co expression network analysis found that the turquoise module had the strongest correlation with DKD (R = −0.58, *P* = 4 × 10^-4^). Thirty-eight overlapping genes of DEGs and turquoise modules were extracted. The identified DEGs were mainly involved in p53 signaling pathway, HIF-1 signaling pathway, JAK − STAT signaling pathway and FoxO signaling pathway between and the control. C-X-C motif chemokine ligand 3 was identified as diagnostic markers of DKD with an area under the receiver operating characteristic curve of 0.735 (95% CI 0.487–0.932). C-X-C motif chemokine ligand 3 was identified as diagnostic biomarkers of DKD and can provide new insights for future studies on the occurrence and the molecular mechanisms of DKD.

## 1. Introduction

Diabetes mellitus is a group of metabolic diseases characterized by hyperglycemia resulting from defects in insulin secretion, insulin action, or both. The prevalence of diabetes is increasing worldwide. The International Diabetes Federation estimates that there are 425 million (18–99 years) people with diabetes worldwide in 2017, which will reach 693 million people by 2045.^[1]^ Diabetic kidney disease (DKD) is a key micro-vascular complication of diabetes that induces a progressive decline in renal function, over 5 stages, leading to kidney failure.^[2]^ At present, DKD is the leading cause of end-stage renal disease worldwide.^[3]^ Among people with diabetes, the development of DKD carries a higher mortality risk.^[4]^

DKD classically identified by the presence of proteinuria in people with diabetes. However, increasing evidence has shown that a significant number of patients with diabetes may have decreased glomerular filtration rate (GFR) without significant albuminuria, known as non-albuminuric DKD.^[5]^ Although both albuminuria and GFR are well-established diagnostic biomarkers of DKD. However, both albuminuric and GFR loss are nonspecific markers of DKD, as they are altered in most chronic glomerulopathies.^[6,7]^ Meanwhile, a number of patients with DKD who do not follow the classic pattern of DKD.^[8,9]^ Given the limitations of current markers, there is the need to identify novel diagnostic biomarkers for DKD.

Recent years, microarray technology and integrated bioinformatics analysis, had been performed to identify novel genes related to DKD.^[10,11]^ For example, the upregulation of the FcER1 gene in DKD patients was found.^[12]^ At present, there are some successful cases of using bioinformatics to screen molecular markers,^[13,14]^ but the research mostly uses a traditional of bioinformatics algorithms, which may lead to excessive data interference and poor reliability of the results. We applied bioinformatics analysis using system biology method combined with machine learning algorithm to investigate candidate diagnostic marks for DKD to improve the accuracy of screening molecules.

Weighted gene co expression network analysis (WGCNA) is a nontraditional data analysis method. The traditional method of analyzing data is to find differentially expressed genes and process each gene separately. WGCNA method classifies genes into several modules according to the similarity of gene expression changes.^[15]^ Compared with classical differential expression gene analysis methods, WGCNA method can greatly reduce the problem of multiple hypothesis testing, reduce the dimension of high-dimensional data and integrate multiple data, such as combining gene expression data with clinical indicators for analysis. However, WGCNA has certain requirements for sample size, such as if the sample size is too small to be < 30, resulting in unstable and non reproducible.

Least absolute shrinkage and selection operator (LASSO) algorithm is a regression method, which can be used to clarify the specific correlation degree of 2 related variables.^[16]^ Compared with traditional Cox regression and logistic regression, Lasso algorithm can reduce the dimension. The WGCNA and LASSO algorithms are widely used in bioinformatics analysis and exhibit an important in clinical application of various fields. Support vector machine (SVM) is a kind of general learning method with small samples. SVM-recursive feature elimination (RFE) is an algorithm that combines SVM with RFE. SVM-RFE belongs to backward search algorithm, which reduces the dimension of space by selecting and eliminating unnecessary features.^[17]^ The advantages of SVM algorithm are suitable for small sample data and are less affected by noise and mainly supports binary classification.

**Figure 1.**
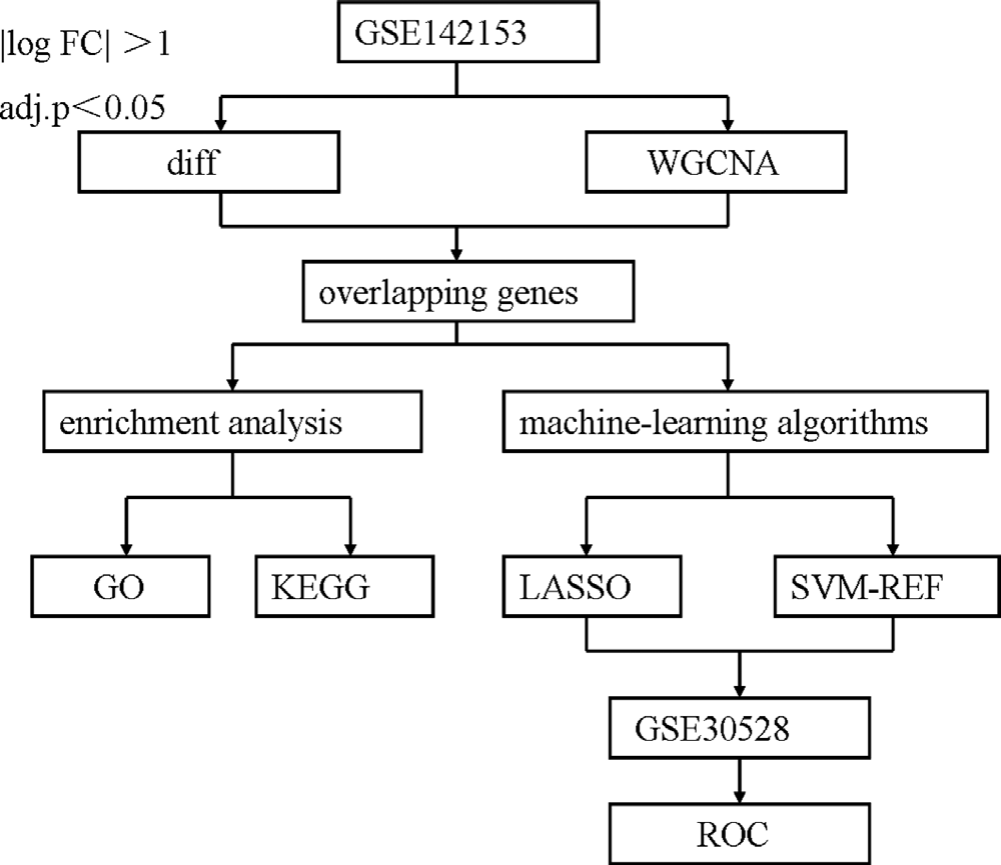
Flowchat. DEGs = differentially expressed genes, GO = gene ontology, KEGG = kyoto encyclopedia of genes and genomes, LASSO = least absolute shrinkage and selection operator, RFE = recursive feature elimination, SVM = support vector machine, WGCNA = weighted gene co expression network analysis.

In order to find biomarkers for the diagnosis of DKD, we downloaded 1 microarray dataset of DKD from the GEO database. Differentially expressed genes (DEGs) analysis and WGCNA were performed between the DKD and controls. Machine learning algorithms were used to filter and identify diagnostic biomarkers of DKD. The study flow is illustrated in Figure 1.

## 2. Materials and methods

### 2.1. Microarray data

We acquired gene expression profile datasets GSE142153 and GSE30528 from the GEO database (https://www.ncbi.nlm.nih.gov/geo/). The GSE142153 dataset included 23 DKD and 10 controls collected from circulating endothelial cells. The GSE142153 dataset included 9 DKD and 13 controls collected from the kidney tissue. The probes were changed into gene symbols based on their probe annotation files.

### 2.2. Data processing and DEG screening

The SVA package was used to preprocess and remove batch effects.^[18]^ The limma package of R (http://www.bioconductor.org/) was used for background correction, normalization between arrays, and differential expression analysis between 23 DKD and 10 control samples from GSE142153 database. Samples with an adjusted false discovery rate *P* < .05 and |log fold change| > 1 were considered as the threshold points for DEGs.

### 2.3. Establishment of gene co expression network

The gene co expression network was established by using WGCNA package of R language. Assume that the gene network obeys the scale-free distribution, and define the gene co expression correlation matrix and the adjacency function formed by the gene network, then calculate the dissimilarity coefficient of different nodes, and build a hierarchical clustering tree according to. Different branches of the clustering tree represent different gene modules. Then the modules related to DKD were selected for further analysis.

### 2.4. Functional enrichment analysis

Overlapping genes between DEGs and correlation modules were extracted. Gene ontology enrichment analyses were performed on the overlap genes between DEGs and correlation modules using the “clusterProfiler” and DOSE packages in R.^[19,20]^ Kyoto encyclopedia of genes and genomes (KEGG) was used to identify the most significant functional terms between the overlapping genes. A gene set was regarded as significantly enriched if a *P* < .05 and false discovery rate < 0.05.

### 2.5. Candidate diagnostic biomarker screening

Two machines learning algorithms were used. The LASSO regression algorithm was carried out using the “glmnet” package in R to identify the genes significantly associated with the discrimination of DKD and normal samples. Set the parameter alpha to 1 and parameter n to 10. SVM-RFE algorithm was employed to select the optimal genes from the meta data cohort.^[21]^ The sampling method adopts cross validation. The overlapping genes between the 2 algorithms were included.

### 2.6. Diagnostic value of feature biomarkers in DKD

To test the predictive value of the identified biomarkers, we generated an ROC curve using the gene data from GSE30528. The area under the ROC curve value was utilized to determine the diagnostic effectiveness in discriminating DKD from control samples.

### 2.7. Statistical analysis

The data were analyzed by R (version 3.6.1) for windows. LASSO regression analysis was carried out using the “glmnet” package, and the SVM algorithm was performed using the e1071 package in R. ROC curve analysis was used to determine the diagnostic efficacy of the diagnostic biomarkers included. A value of *P* < .05 (2-sided test) was considered significant.

## 3. Results

### 3.1. Identification of DEGs in DKD

**Figure 2.**
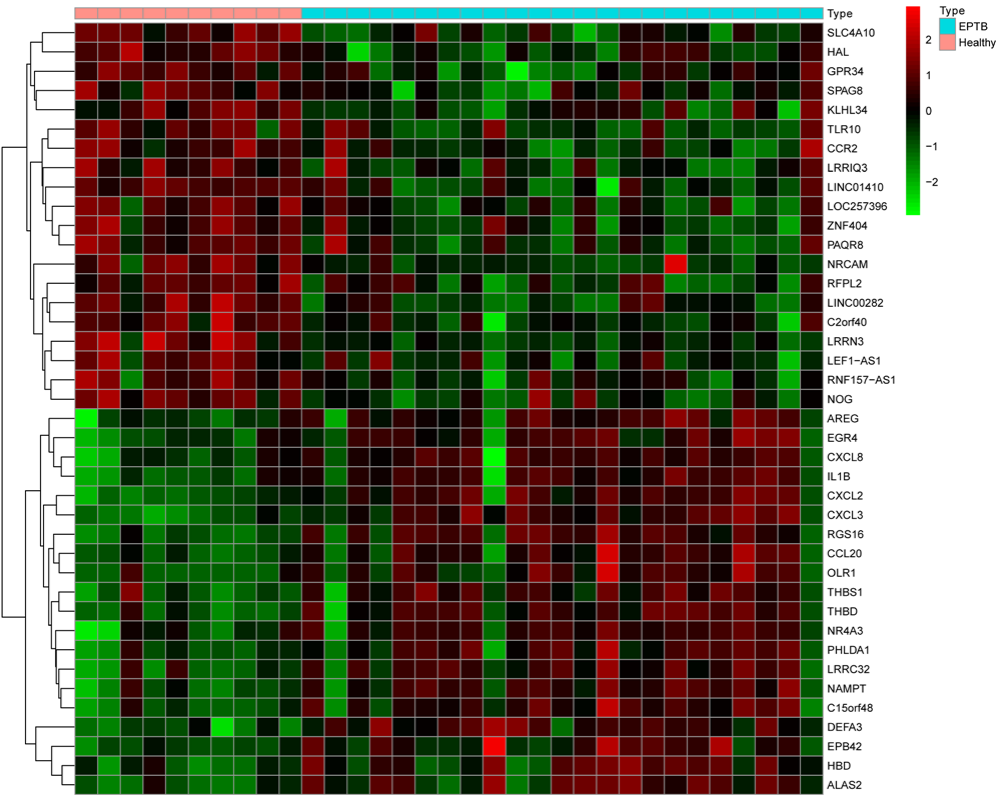
Different expressed genes between diabetic kidney disease and control samples.

Data from a total of 23 DKD and 10 control samples from GSE142153 were analyzed in this study. The average age of the 2 group are 60 and 36.6 years respectively. The average eGFR of the 2 group are 68.2 and 82 mL/minutes/1.73 m^2^ respectively. The average BMI of the 2 group are 35.7 and 25.8 kg/m^2^. The DEGs of the data were analyzed using the limma package after removing the batch effects. A total of 110 DEGs were obtained: 64 genes were significantly upregulated and 46 genes were significantly downregulated (Fig. 2).

### 3.2. Gene co expression network analysis

**Figure 3.**
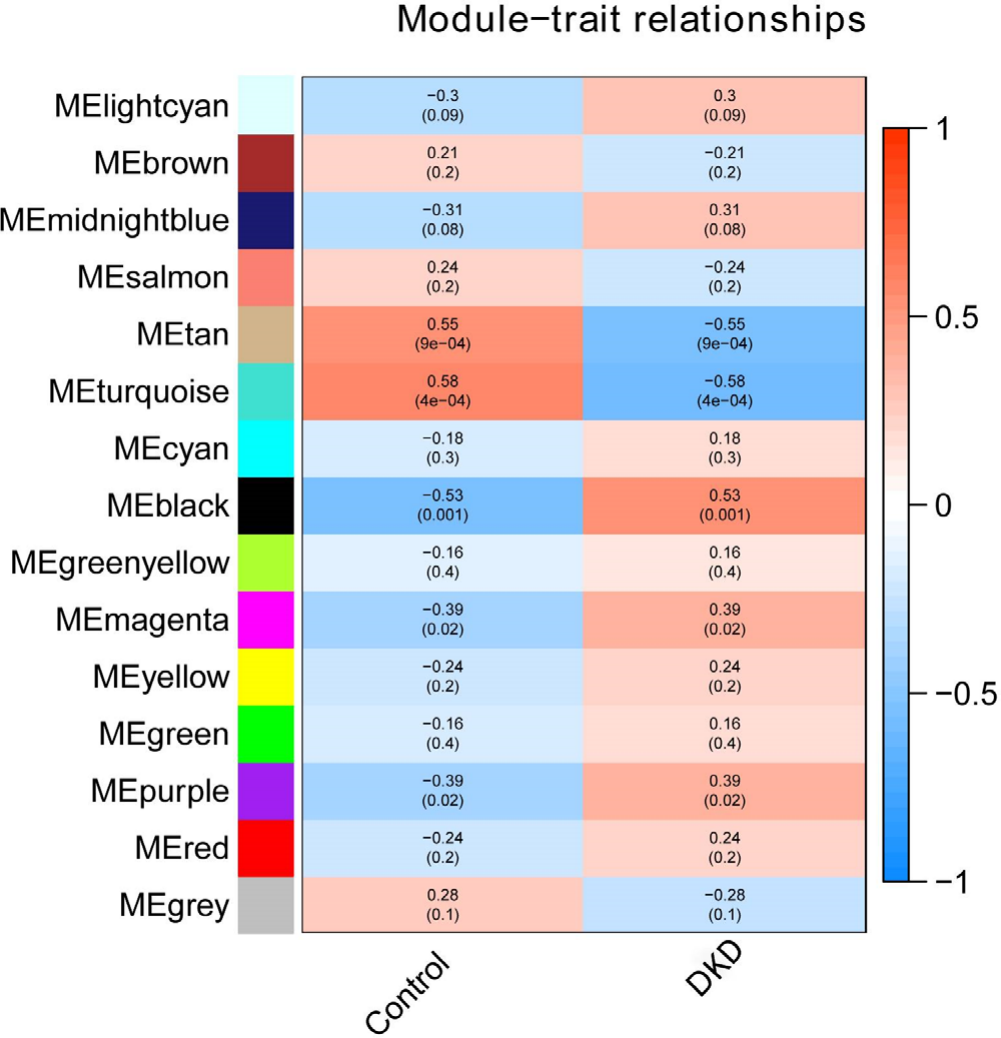
Module-trait correlations and *P* values. Each cell reports the correlation (and *P* value) resulting from correlating module eiggengenes (rows) to traits(columns), the table is color-coded by correlation according to the legend (red indicates positive correlation while blue indicates negative correlation). DKD = diabetic kidney disease.

The gene co expression network was analyzed using the WGCNA package after filtering the genes with low expression and little change in expression, Genes that meet 2 conditions simultaneously are filtered out: The range of expression levels of the gene in the sample (the difference between the highest and lowest expression levels) is less than the median of the range of expression levels corresponding to all genes; The average expression level of this gene in all samples is less than the median of the corresponding expression level of all genes. After filtering, 6054 genes were used for WGCNA and 15 modules were obtained: black (1476), brown (869); cyan (99), green (475), greenyellow (164), gray (2), lightcyan (95), magenta (190), midnightblue (64), purple (188), red (387), salmon (102), tan (135), turquoise (1256), and yellow (552). The correlation between the intrinsic gene of the module and DKD was calculated to find the relevant gene module. It was found that the turquoise module had the strongest correlation with DKD (R = −0.58, *P* = 4 × 10^-4^) (Fig. 3).

### 3.3. Go analysis and KEGG pathway enrichment analysis

**Figure 4.**
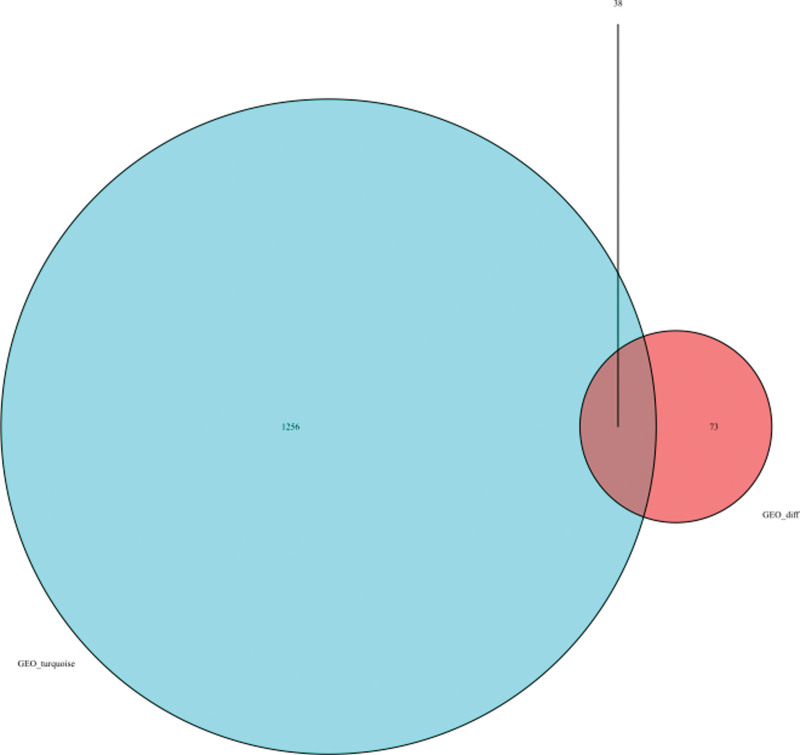
Venn diagram.

**Figure 5.**
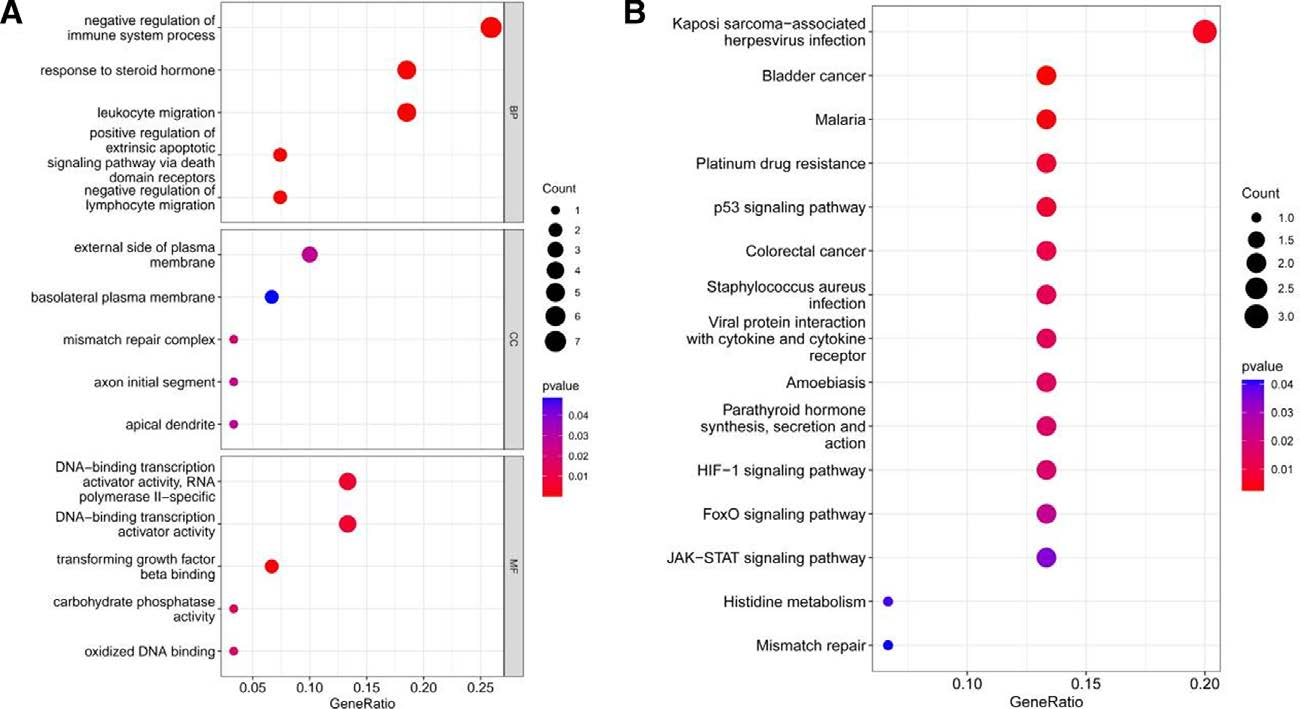
GO analyses and KEGG pathways enriched in the overlapping genes of DEGs and Turquoise modules. BP =biological process, CC =cellular component, DEGs = differentially expressed genes, GO = gene ontology, KEGG = Kyoto encyclopedia of genes and genomes, MF =molecular function.

38 overlapping genes of DEGs and turquoise modules were extracted (C-X-C motif chemokine ligand 3 [CXCL3], DEFA3, MSH2, LINC00282, SLC4A10, OSR2, PFKFB3, TNFAIP8L2, VSIG1, HAL, FAM46C, LINC01410, NRCAM, CD200R1, IL10, PAQR8, MAFB, SIRT4, GFOD1, ZNF781, NUDCD1, LRRC32, ZNF404, SGK223, GCSAM, ATF3, LOC257396, THBS1, ATP6V0E2-AS1, CDKN1A, HAB1, MAFF, IGIP, HAS1, FOXL2, LRRIQ3, IGKV1-5, and HCAR3) (Fig. 4). Gene ontology analyses were conducted to investigate the function of 38 overlapping genes. The results of go analysis showed that there were 421 biological processes items, 5 cell composition items and 29 molecular function items. The first 5 items were visualized respectively. Biological process analysis includes important pathways such as negative regulation of immune system process, response to steroid hormone and leukocyte migration; cell composition analysis is mainly manifested in external side of plasma membrane, basolateral plasma membrane and mismatch repair complex; The expression of molecular function is mainly DNA -binding transcription activator activity, RNA Polymerase II- specific, DNA- binding transcription activator activity, etc (Fig. 5A). The results showed that 38 differential genes were mainly involved in immune regulation, plasma membrane and cell membrane. The KEGG results demonstrated that the enriched pathways mainly involved p53 signaling pathway, HIF-1 signaling pathway, JAK − STAT signaling pathway and FoxO signaling pathway (Fig. 5B).

### 3.4. Identification and validation of diagnostic feature biomarkers

**Figure 6.**
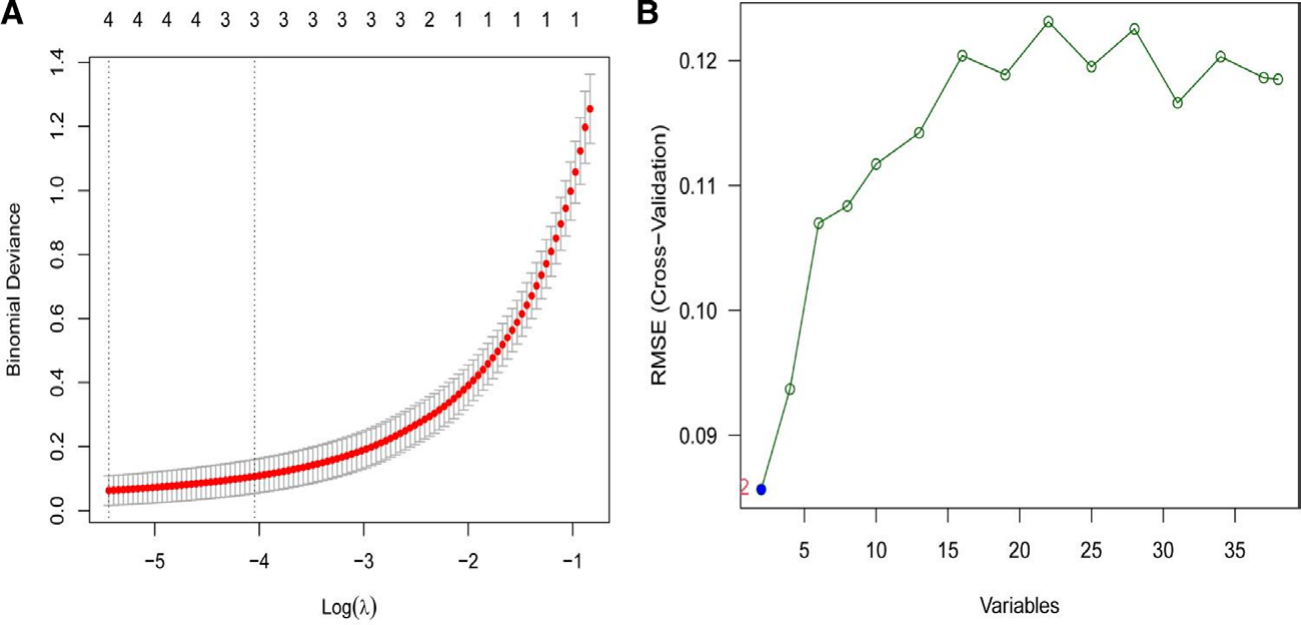
Screening process of diagnostic biomarker candidates for diabetic kidney disease diagnosis. (A) Tuning feature selection in the least absolute shrinkage and selection operator model. (B) A plot of biomarkers selection via support vector machine-recursive feature elimination algorithm.

Two different algorithms were used to screen potential biomarkers. The 38 overlapping genes of DEGs and Turquoise modules were narrowed down using the LASSO regression algorithm, resulting in the identification of 4 variables as diagnostic biomarkers for DKD (Fig. 6A). A subset of 2 features among the 38 overlapping genes was determined using the SVM-RFE algorithm (Fig. 6B). The 2 overlapping features (CXCL3 and LINC00282) between these 2 algorithms were ultimately selected. Therefore, the 2 identified genes were used to establish the diagnostic model using a logistic regression algorithm in the meta data cohort.

### 3.5. Diagnostic effectiveness of feature biomarkers in DKD

**Figure 7.**
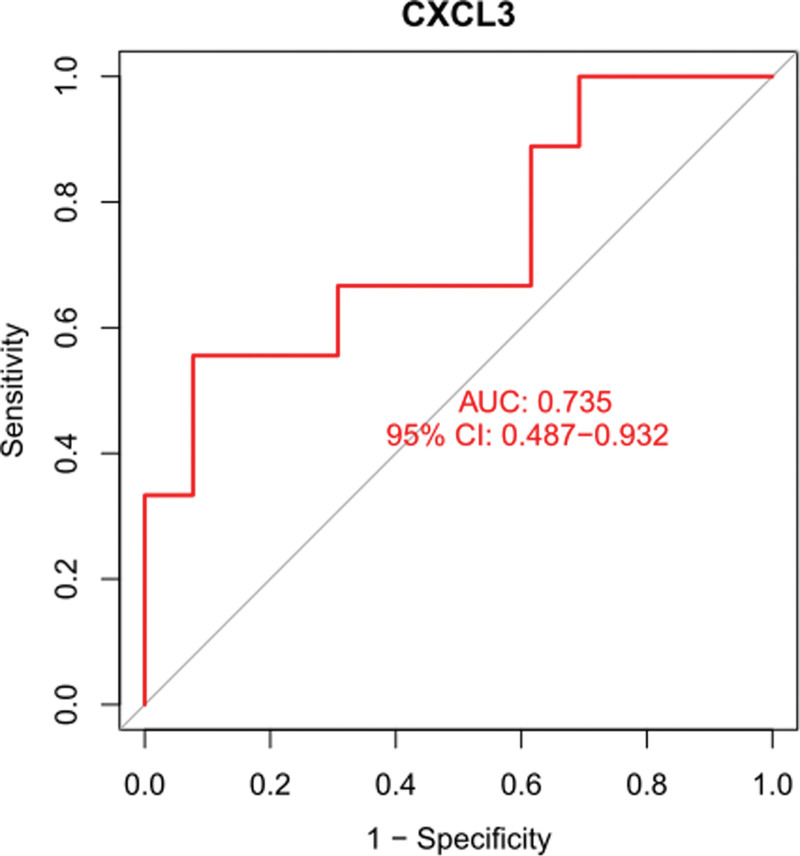
The receiver operating characteristic curve of the diagnostic effectiveness of CXCL3. CXCL3 = C-X-C motif chemokine ligand 3.

The diagnostic ability of CXCL3 in discriminating DKD from the control samples demonstrated a favorable diagnostic value, with an area under the receiver operating characteristic curve of 0.735 (95% CI 0487–0.932). (Fig. 7). However, we did not find any expression of LINC00282 in the GSE30528 dataset.

## 4. Discussion

DKD is one of the most common diabetic complications, as well as the leading cause of chronic kidney disease and end-stage renal disease around the world. Because of the lack of an effective early diagnosis, patients with DKD often lose the chance to benefit from treatment, resulting in poor outcomes.^[22]^ At present, urinary albumin-to-creatinine ratio and eGFR are well-established diagnostic biomarkers of DKD.^[23]^ However, diagnosing DKD also faces challenges associated with both albumin-to-creatinine ratio and eGFR loss are nonspecific markers of DKD, and a number of patients with DKD who do not follow the classic pattern of DKD.^[8,9]^ Therefore, researchers are increasingly searching for novel diagnostic biomarkers of DKD.

Recently, mRNAs and microRNAs have emerged as promising biomarkers in DKD.^[13,14,24,25]^ For example, let-7b-5p and miR-21-5p could serve as biomarkers to predict the risk of ESKD in T1DM, where the elevated expression of the let-7b-5p and miR-21-5p are independent risk factor for ESKD. In particular, let-7c-5p and miR-29a-3p were independently associated with more than a 50% reduction in the risk of rapid progression to ESKD in T1DM.^[24]^ Another study of patients with T1DM without albuminuria revealed that 18 microRNAs were associated with the development of albuminuria and 9 of them were used to define a gene signature for microalbuminuria.^[25]^ However, the research mostly uses a traditional of bioinformatics algorithms, which may lead to excessive data interference and poor reliability of the results. To improve the accuracy of screening molecules, we applied bioinformatics analysis using system biology method combined with machine learning algorithm to investigate candidate diagnostic marks for DKD.

As far as we know, this is the first retrospective study to identify diagnostic biomarkers in patients with DKD by GEO datasets with WCGNA and machine learning algorithm. We collected 1 cohort from the GEO datasets and conducted an integrated analysis of the data. A total of 110 DEGs were identified, including 64 upregulated genes and 46 downregulated genes. Turquoise module had the strongest correlation with DKD with gene co expression network analysis. Thirty-eight overlapping genes of DEGs and turquoise modules were found. The results of enrichment analyses indicated that diseases enriched by the overlapping genes were mainly associated with immune regulation, plasma membrane and cell membrane. These findings are in general agreement with the previous finding that an inflammatory response involving leukocytes participates in the pathogenesis of DKD.^[26]^

The KEEG results demonstrated that the enriched pathways generally involved in p53 signaling pathway, HIF-1 signaling pathway, JAK − STAT signaling pathway and FoxO signaling pathway. Ma Z et al^[27]^ found that a positive correlation between p53 signaling pathway and renal fibrosis from patients with diabetes. At the same time, they found that p53 microRNA-214/ULK1 axis signaling pathway participates in the occurrence of DKD by inhibiting renal tubular autophagy. Guo W et al^[28]^ found SIRT1/P53/NRF2 pathway modulates the pathogenesis of DKD. SRT2104, which is a novel, first-in-class, highly selective small-molecule activator of SIRT1 can enhanced renal SIRT1 expression and activity, deacetylated P53, and activated NRF2 antioxidant signaling, providing remarkable protection against the DM-induced renal oxidative stress, inflammation, fibrosis, glomerular remodeling, and albuminuria in the diabetic mice models.^[29]^ Serum HIF-1α may be involved in DKD process through inflammation, angiogenesis, and endothelial injury.^[30]^ However, the signal pathway is unknown. At present, we found that in mesangial cells, elevated glucose levels induce HIF activity by a hypoxia-independent mechanism. Elevated HIF activity in glomerular cells promotes glomerulosclerosis and albuminuria, and inhibition of HIF protects glomerular integrity. However, tubular HIF activity is suppressed and HIF activation protects mitochondrial function and prevents development of diabetes-induced tissue hypoxia, tubulointerstitial fibrosis and proteinuria.^[31]^Therefore, We need further research. The JAK-STAT pathway transmits signals from extracellular ligands, including many cytokines and chemokines as well as growth factors and hormones, directly to the nucleus to induce a variety of cellular responses.^[32]^ Gene and protein expression studies of kidney biopsies from people with early- and late-stage DKD have shown increased activation and expression of the JAK-STAT signaling pathway across the spectrum of DKD.^[33]^ Inhibitors of JAK/STAT pathways are promising therapeutic options to improve the renal outcome of patients with DKD, but appropriate clinical trials are necessary.^[34]^

Based on 2 machine learning algorithms, 2 diagnostic markers were identified. CXCL3 is a member of the CXC subfamily of chemokines produced by inflammatory cell. It mainly recruits and activates a variety of cells expressing CXC chemokine receptor 1 and 2, participates in the regulation of cell migration, invasion and angiogenesis.^[35]^ At present, the research on CXCL3 mainly focuses on tumor immunity.^[36]^ Blocking CXCL3 signal transduction pathway can inhibit the pathophysiological processes such as cell migration, invasion, angiogenesis, tumorigenesis and fibrosis, which may become a potential prevention and treatment target for a variety of diseases. We need further research to understand the role of CXCL3 in DKD.

LINC00282 is also known as transmembrane protein 272 (TMEM272), which been predicted to be integral component of membrane (https://www.ncbi.nlm.nih.gov/gene/283521). At present, the role of LINC00282 in the process of DKD is not clear. There is a differential expression of LINC00282 in blood samples of patients with DKD, but the expression of LINC00282 is not detected in kidney tissue. Therefore, further research is needed.

The limitations of this study should be acknowledged. First, the study was retrospective; thus, important clinical information was not available. Second, the relatively small number of cases in GSE142153 should be considered a limitation. In addition, the biomarker profiles in the blood cell were obtained from the datasets, and their reproducibility should be further validated. Prospective studies with larger sample sizes should be conducted to validate our conclusions.

## 5. Conclusion

In summary, CXCL3 was identified as diagnostic biomarkers of DKD and can provide new insights for future studies on the occurrence and the molecular mechanisms of DKD.

## Acknowledgments

The authors acknowledge the Gene Expression Omnibus (GEO) database for providing data of DKD available.

## Author contributions

**Data curation:** Ya Nan Wang.

**Methodology:** Ya Nan Wang.

**Resources:** Hua Wei Jin.

**Supervision:** Wen Fang Xu.

**Writing – original draft:** Qian Gao.

**Writing – review & editing:** Qian Gao.
